# Substance use and risky sexual behaviours among street connected children and youth in Accra, Ghana

**DOI:** 10.1186/1747-597X-9-45

**Published:** 2014-11-27

**Authors:** Kwaku Oppong Asante, Anna Meyer-Weitz, Inge Petersen

**Affiliations:** Discipline of Psychology, School of Applied Human Sciences, University of KwaZulu-Natal, Howard College Campus, Durban, 4041 South Africa; Department of Psychology, Regent University College of Science & Technology, Accra, Ghana

**Keywords:** Aggregation of HIV risk behaviour, Ghana, Street youth, Substance use

## Abstract

**Background:**

Research on street children and youth has shown that this population is at high risk for substance use. Though risky sexual behaviours have been investigated and widely reported among street youth in resource constrained-settings, few studies have explored the relationship between substance use and other risk behaviours. This study was therefore conducted to examine the association between substance use and risky sexual behaviours among homeless youth in Ghana.

**Method:**

A cross-sectional survey of a convenient sample of 227 (122 male and 105 female) street connected children and youth was conducted in Ghana in 2012. Using self-report measures, the relationship between substance use and risky sexual behaviours was examined using logistic regression.

**Results:**

Substance use was relatively high as 12% and 16.2% reported daily use of alcohol and marijuana respectively. There were age and sex differences in substance use among the sample. As compared to males, more females had smoked cigarettes, used alcohol and marijuana. While alcohol use decrease with age, marijuana use on the other hand increases with age. Results from multivariate analysis revealed that having ever drunk alcohol and alcohol use in the past one month were independently associated with all the four indices of risky sexual behaviour (ever had sex, non-condom use, multiple sexual partners and survival sex). Both marijuana use and smoking of cigarettes were associated with having ever had sex, multiple sexual partners and survival sex. Other drug use was independently associated with non-condom use.

**Conclusion:**

Substance use seems to serve as a possible risk factor for sexual risk behaviours among homeless youth. Harm reduction interventions are needed to prevent street children and youth from engaging in substance use and risky sexual behaviours. Such programmes should pay special attention to females and younger children who are highly susceptible to the adverse conditions on the street.

## Background

The phenomenon of homeless/street children is global, with the streets throughout the world are home to millions of children [1]. Circumstances on the street render children and youth vulnerable to various kinds of psychological problems and health risks [2]. Their lifestyles are characterized by a high rate of sex trade [3–5] and substance abuse and misuse [6, 7] which places them at high risk for STI, including HIV infection [8, 9].

The type and prevalence rate of substances used varies remarkably according to context and geographical location. In a comprehensive review, [10] reported that street youth in high income countries usually use injecting drugs and other substances that are not commonly used by homeless adolescents from low and middle resourced countries (LMIC). A study among street children in Egypt indicated that 62% of the participants reported substance use, with the highest substance use being alcohol consumption (35%) with only 3% indicating injection drug use [4]. Similarly, [11] found that eighty-two percent of street children in the Democratic Republic of Congo (DRC) used marijuana, 63.5% used alcohol and 3.8% used cocaine [11]. A similar pattern of substance use was also reported among street youth in Kenya, where lifetime and current substance use was 74% and 83% respectively [12].

Substance use among homeless youth has been found to be influenced by factors such as gender, age, duration of homelessness and social networks (e.g. peer influence). Research has indicated that more males than females use alcohol, marijuana, cocaine and inhalants (i.e. glue) [6, 9, 11, 13, 14]. Further, evidence shows that although differences in the choice of substances exist, the prevalence of substance use disorders is similar among males and females [15]. Another factor that influences substance use is age [4, 9]. In a study of drug use among young and older homeless youth, [9] found that younger participants (less than 21 years) where more likely to be engaged in excessive drinking of alcoholic beverages, while older participants (21 years and more) were more likely to be involved in heroine, crack and injecting drug use. The number of years lived on the street influences substance use [7, 12, 16]. Similarly, [17] reported from their cross-national study that the length of homelessness was associated with higher rates of substance misuse and psychiatric disorders.

Substance use also increases the likelihood that individuals will engage in risky sexual behaviours, for example, non-condom use and multiple sexual partner patterns which put them at risk of STIs and HIV/AIDS [4, 10, 11]. A study conducted among 280 street youth in Ethiopia indicated that 77% of the sample, who indicated they were sexually active at the time of the study, did not use condoms during their last sexual activity [18]. The sexual behaviour trends of street youth show that not only are street youth not protected sexually, but that they also have multiple sexual partners [4, 8, 9, 11, 19, 20]. A study conducted by [8] on street youth in Ethiopia revealed that 62% of their sample were sexually active and 97% of the sexually active had multiple sexual partners. The same study further showed that 80.5% of the street youth used condoms inconsistently, and the length of stay on the street was also associated with inconsistent condom use [8]. Reasons for non-condom use among street youth include not been able to buy condoms, drug use and negligence as well as belief that it decreases pleasure, and difficulty in using it in the “heat of the moment” [5, 8, 11].

Closely related to having multiple sexual partners and inconsistent condom use, is transactional sex, especially for female street youth (which includes sex for money and sometime for protection on the street) [4, 20–22]. Among homeless children in Egypt, [4] found that 25% of the girls had sold sex to males, while [21] reported in a study in Tanzania that those aged between the ages of 11–17 years engaged in commercial sex.

Evidence from Ghana indicates that homeless youth is growing in cities such as Accra. Headcounts of street children ranged from 35,000 in 2009 [23], 2010) to 90,000 in 2013 [24]. Despite this increasing number of homeless children and youth, the prevalence of substance use and abuse, and the relationship between substance use and sexual risk behaviours has not been examined in Ghana. There is also sparse literature on age and gender differences regarding substance use among homeless children and youth in Ghana. Previous studies conducted among homeless children and young adults in Ghana, have focused primarily on economic, social and cultural causes of homelessness, their engagement in risky sexual behaviours and the prevalence of STI including HIV and AIDS [5, 20, 25–28].

In the context of this gap in knowledge, the aims of this study aims to determine i) the prevalence of substance use and misuse ii) age and sex differences in substance use and iii) the relationship between substance use and risky sexual behaviours among a sample of street connected children and youth in the Central Business District (CBD) of Accra, Ghana.

## Method

### Design and participants

A cross-sectional study was conducted among homeless children and adolescents in the CBD of Accra, Ghana where the second largest number of street children in Ghana can be found [23] (Catholic Action for Street Children [CAS], 2010). A previous study among street children in Accra indicated that they congregate in places such as markets, bus and train stations [29] (Hatløy and Huser, 2005), all within the CBD of Accra. Participants were eligible to participate in the study if they 1) self identify themselves as being homeless (i.e. live alone or with a group of other youth on the street, have no stable place of residence and experience movements in short periods of time 2) able to give assent or consent to participate in the study and 3) not experiencing acute intoxication or obvious mental health problems, or problematic behaviours. Out of 265 participants approached, 227 agreed to participate in the study. This represents a response rate of approximately 86%. The sample was made up of 122 males and 105 females homeless children and youth, with ages ranging from 8–19 years with a mean age of 12.58 (SD = 2.51).

### Procedures

Two research assistants who were fluent and knowledgeable of the language spoken by the street youth were recruited and trained. The research participants were approached at specific designated places and asked whether they would be willing to participate in the study. Those who agreed were informed about the objectives of the study and ethical principles i.e. that their participation was voluntary and that they could withdraw from the study without any consequences to them during the course of the study, that the information would remain confidential and anonymous and be used for research purposes. The participants gave oral consent and or assent to participate in the study. As the standard practice with self-reliant youth populations not in contact with or otherwise supervised by an adult guardian [30], the researchers considered this as an appropriate means to seek informed consent from the participants. The data was collected through an interviewer-administered questionnaire (as a result of low level of education) and most of the participants listened attentively to the questions. It took an average of 30 minutes to administer the full questionnaire and data collection lasted for 8 weeks. The majority of the participants were interviewed in Twi and Ga (two predominant local languages spoken in Accra, Ghana). Each participant was compensated with a voucher worth approximately US$2.00 as a reward for volunteering participation in the study. While this amount may seem insignificant, this would have enabled the youth to buy a daily meal. None of the participants expressed the need for psychological service although they were told of the availability of a psychologist should they require such a service. Ethical approval to conduct the study was granted from the Department of Social Welfare, Accra, Ghana and the Human and Social Science Ethics Committee (Ethical Approval number: HSS/1144/012D) of the University of KwaZulu-Natal, Durban, South Africa.

### Survey instrument

The survey questionnaire used in this study was modelled on the South African Youth Risk Behaviour Survey [31]. The development was also informed by a pilot study conducted among 26 homeless youth in a similar but different location. This was done to determine the appropriateness of the questions asked and also to ensure that it elicits relevant responses. The survey questions addressed demographic characteristics (e.g. sex, age, years of living on the street, highest level of education and reasons which led them to live on the street), substance use (5 items; Cronbach’s α = 0.85) and sexual risk behaviours (4 items) in the last one month. For the purpose of this study, substance use refers to the use of alcohol, marijuana, smoking of cigarette and other drug use such as cocaine, glue or heroin. The questions on substance use includes “Have you ever drunk an alcoholic beverage”, “Have you used alcohol in the past month” “Have you ever smoked a cigarette”, “Have you ever used marijuana” and “Have you ever used any drugs such as cocaine, glue or heroin”. Risky sexual behaviours covered issues such as ever had sexual intercourse, non-condom use, multiple sexual partners and exchange of sex for other resources and or for protection. The response format to these items on the questionnaire were in the form of yes = 1 and no = 0. Total scores were not calculated from these items, as the study was interested in the specific issues that each single item covered. Single items were therefore used in the analyses.

### Data analyses

Data was analysed by means of the Statistical Package for the Social Sciences (SPSS) version 21.0 for Window (IBM SPSS, Inc., Chicago, IL, USA). Three (3) age groups (7–10 years, 11–13 years and 14 years and older were created to ensure equal variance between the ages. These categorizations closely correspond to late childhood, early and late adolescence. These stages of development have distinctive features and abilities [32, 33]. Descriptive statistics was used to examine demographic information, the rates of substance use and HIV risk behaviours. Chi-square (χ^2^) was used to examine the association between substance use, risky sexual behaviours and, gender and age. There were four dependent variables namely ever had sexual intercourse, inconsistent condom use, multiple sexual partners and engagement in survival sex. Univariate and multivariate logistic regression analyses were conducted to determine the substance use predictors of risky sexual behaviours. The results from the regression analyses are presented as odds ratio (OR) and 95% confidence interval (CI). All analyses were two-tailed, and a *p*-value of less than 0.05 was considered statistically significant.

## Results

### Demographics characteristics of the sample

Males comprised approximately 53.7% of the sample size with the remaining 46.3% being females. The majority of the participants had migrated from 6 regions and had come to Accra either alone or with a help of a family member, and over 80% of the sample were 14 years old or younger. Over 59% of the participants indicated that poverty was the main reason for being homeless and about 25% had been abused (both physically and sexually). Over half (58.9%) of the participants had up to primary education level, and about 43% had lived on the street for 3–8 years. Significantly more females (19.8%) reported sexual abuse as the cause of leaving home than males (2.5%), χ^2^ (1, 223) =22.87, p < 0.001 (Table 1).

**Table 1:** **Demographic profile of the sample**

Characteristics	***N***	%
*Gender*		
Male	122	53.7
Female	105	46.3
Ages (*M* = 12.58, *SD* = 2.51)		
8–10 years	50	22.4
11–14 years	129	57.9
15 years and over	44	19.7
*Previous level of education*		
No formal education	69	30.5
Primary school (Grade 1–6)	133	58.9
Junior secondary school (Grade 7–9)	24	10.6
*Years living on the street*		
*< 1 year*	26	11.6
1-2 years	101	45.1
3-5 years	69	30.8
5 years and more	28	12.5
*Reasons for being homeless*		
Family poverty	132	59.2
Dysfunctional problems	15	6.7
Maltreatment: Sexually abused	23	10.3
Maltreatment: Physical abused	34	15.3
Divorce	12	5.4
Other reasons	7	3.1

*N* = Number, % = Percentage of N, *M* = Mean, *SD* = Standard Deviation.

### Sex and age group differences in substance use

Substance use was relatively high among the study sample. The results as presented in Table 2 showed that over 66.2% of the sample reported to have ever smoked cigarettes, 81.3% reported having used alcoholic beverages, out of which 70.1% had used alcohol in the preceding month to the study. Approximately 72% indicated to have ever smoked marijuana, and about12.0% and 16.2% of the participants reported daily alcohol and marijuana use respectively (not shown Table 2). A statistical significance sex difference was noted in substance use as compared to boys, females were more likely to have smoked cigarette (73.4% females vs. 60.3% males; *p* < 0.05), drunk alcoholic beverage (90.1% females vs. 73.7%males; *p* = 0.002), and more likely to have ever used marijuana (79.0% females vs. 65.5% males; *p* < 0.05). Conversely, more males were likely to have used other drugs, i.e. cocaine, glue or heroin (30.2% males vs. 15.1% females; *p* = 0.010).

**Table 2:** **Distribution of sexual behaviours and substance use variables by gender**

Sexual behaviour pattern	Total	Males	Females	***p***-values^a,b^
	(N = 227)	(N = 122)	(N = 105)	
Had sex in the last one month (N = 151)^c^				**0.038**
Yes	69.3	63.2	76.2	
No	30.7	36.8	24.8	
Condom use at last sexual activity (N = 37)^c^				0.225
Yes	17.1	14.5	20.2	
No	83.9	83.5	79.8	
Multiple sexual partners (N = 118)^c^				
Yes	54.9	50.0	60.2	0.119
No	55.1	50.0	39.8	
Survival sex (N = 115)^c^				**0.010**
Yes	53.0	44.8	62.4	
No	47.0	55.2	37.6	
Smoking (N = 139)^c^				**0.047**
Yes	66.2	60.3	73.4	
No	33.8	39.7	26.6	
Ever drunk alcoholic beverage (N = 178)^c^				**0.002**
Yes	81.3	73.7	90.1	
No	18.7	26.3	9.9	
Had used alcohol in past one month (N = 147)^c^				0.162
Yes	70.1	66.7	75.5	
No	29.9	33.3	24.5	
Marijuana use (N = 149)^c^				**0.030**
Yes	72.0	65.5	79.0	
No	28.0	34.5	21.0	
Other drug use (N = 209)^c^				**0.010**
Yes	23.4	30.2	15.1	
No	76.6	69.8	84.9	

^a^Statistical significant *p* values in bold.

^b^Missing values were excluded in the statistics test.

^c^N = number of cases used in the analysis.

The results in Table 3 revealed age-group differences in relation to substance use. Alcohol use in the last month varied by age, *p* = 0.002. Street youth aged (11–13 years) reported using alcohol (81.1%) more than those the (14–19) years (71.4%) and (7–10) year olds (52.3%) in the last month. Participants aged 11–13 years (92.6%) were more likely to have reported ever drinking alcohol than those aged 14–19 years (83.6%) and 7–10 years (59.6%), *p* < 0.001.These findings therefore suggest that early adolescents were more likely to have ever drunk and use alcohol than both late childhood and late adolescents in the study.

**Table 3:** **Distribution of sexual behaviours and substance use variables by age groups**

Sexual behaviour pattern	Total	7 –10 years	11–13 years	14–19 years	p-values^a,b^
	N = 223	N = 50	N = 129	N = 44	
Had sex in the last one month (N = 151)^c^					**0.001**
Yes	69.8	51.1	79.4	61.9	
No	30.2	48.9	20.6	38.1	
Condom use at last sexual activity (N = 37)^c^					0.385
Yes	17.4	10.6	19.2	19.5	
No	82.6	89.4	80.8	80.5	
Multiple sexual partners (N = 118)^c^					**0.001**
Yes	55.2	34.0	34.1	45.6	
No	44.8	66.0	65.9	54.4	
Survival sex (N = 115)^c^					**0.004**
Yes	53.3	31.9	58.4	61.9	
No	46.7	68.1	41.6	38.1	
Smoking (N = 138)^c^					0.362
Yes	67.0	58.7	68.2	70.8	
No	33.0	41.3	32.8	29.2	
Ever drunk alcoholic beverage (N = 177)^c^					**< 0.001**
Yes	82.3	59.6	92.6	83.6	
No	17.7	40.4	7.4	16.4	
Had used alcohol in past one month (N = 146)^c^					**0.002**
Yes	71.6	52.3	81.1	71.4	
No	18.4	47.7	18.9	28.6	
Marijuana use (N = 148)^c^					**0.042**
Yes	72.5	57.1	76.0	77.3	
No	17.5	42.9	24.0	22.7	
Other drug use (N = 206)^c^					
Yes	23.3	45.7	12.2	31.6	**< 0.001**
No	76.7	54.3	87.8	68.4	

^a^Statistical significant *p* values in bold.

^b^Missing values were excluded in the statistics test.

^c^N = number of cases used in the analysis.

The results further shows that the use of marijuana increases with age, *p* < 0.05. Late adolescents (14–19 years) were more likely to have used marijuana (77.3%), followed by early adolescents (11–13 years) (76.0%) and late childhood (7–10 years) (57.1%). The majority of the late adolescents (14–19 years) (67.6%) indicated to have tried quitting smoking more often than both early adolescents (11–13 years) (42.9%) and late childhood (7–10 years) (50.0%). This observed difference was found be statistically significant, *p* = 0.008.

### Association between substance use and sexual risk behaviours

Inconsistent condom use was very high (82.9%) and over 53% and 54% of the participants had engaged in survival sex and had multiple sexual partners respectively. In the logistic regression analyses (Table 4), after controlling for socio-demographic factors, alcohol and marijuana use were independently associated with ever had sexual intercourse. Homeless youth who use alcohol were about six times more likely to have had sexual intercourse compared to non-alcohol users (OR = 5.6, 95% CI = 1.3–24.3). Also marijuana users were about eleven times more likely to be sexually active than their counterparts who do not use marijuana (OR = 10.9, 95% CI = 3.1–38.1). Furthermore, marijuana use among the sample in the study was associated with having multiple sexual partners (OR = 16.6, 95% CI = 4.5–60.6) and engage in survival sex (OR = 8.3, 95% CI = 2.5–28.4). This result implies that that homeless youth who were using marijuana were seventeen and eight times more likely to have multiple sexual partners and engagement in some form of prostitution respectively. The results further show that inconsistent condom use in previous sexual activity was associated with ever drinking alcohol (OR = 22.2, 95% CI = 1.4–34.0) and other drug use (OR = 13.0, 95% CI = 4.6–37.3). These results suggest that street adolescents who have ever drunk alcohol and used alcohol in the past month were independently associated with all the four indices of risky sexual behaviour (ever had sex, non-condom use, multiple sexual partners and survival sex).

**Table 4:** **Odds ratio and their 95% confidence interval (CI) of risky sexual behaviours by substance use among homeless youth in multivariate analyses**

Health Behaviour	Had sexual intercourse	Inconsistent condom use	Multiple sexual partners	Survival sex	Had sexual intercourse	Inconsistent condom use	Multiple sexual partners	Survival sex
	Model 1	Model 1	Model 1	Model 1	Model 2*	Model 2*	Model 2*	Model 2*
	OR (95% CI)	OR (95% CI)	OR (95% CI)	OR (95% CI)	OR (95% CI)	OR (95% CI)	OR (95% CI)	OR (95% CI)
Smoking								
No	1.0	1.0	1.0	1.0	1.0	1.0	1.0	
Yes	**16.9(8.1–34.9)**	1.5(0.7–3.4)	**8.0(4.1–15.9)**	**5.3(2.8–10.0)**	**12.3(5.6–26.9)**	- - - -	**5.6(2.7–12.1)**	**3.2(1.8–7.6)**
Ever drunk alcohol								
No	1.0	1.0	1.0	1.0	1.0	1.0	1.0	1.0
Yes	**16.8(7.1–39.8)**	**10.1(1.3–76.0)**	**17.1(5.8–50.3)**	**11.7(4.4–31.3)**	**14.4(5.5–37.5)**	**7.8(2.0–59.3)**	**12.9(4.1–41.2)**	**9.4(2.9–30.6)**
Had alcohol in past month								
No	1.0	1.0	1.0	1.0	1.0	1.0	1.0	1.0
Yes	**24.9(11.4–54.3)**	**4.9(1.4–16.8)**	**13.6(6.3–29.4)**	**10.8(4.7–25.1)**	**22.2(9.3–53.6)**	**3.1(1.1–15.1)**	**11.4(5.2–25.6)**	**8.8(3.4–18.9)**
Marijuana use								
No	1.0	1.0	1.0	1.0	1.0	1.0	1.0	1.0
Yes	**31.2(13.6 –71.5)**	2.18(0.8–5.6)	**22.0(9.1–52.9)**	**9.3(4.4– 19.6)**	**24.0(9.7–59.8)**	- - - -	**19.4(7.0–50.5)**	**7.5(3.4–16.2)**
Other drug use								
No	1.0	1.0	1.0	1.0	1.0	1.0	1.0	1.0
Yes	0.3(0.2 – 0.7)	**3.9(1.8 – 8.2)**	0.3(0.1 – 0.6)	0.6(0.3 – 1.1)	- - - -	**3.1(1.4–7.7)**	- - - -	- - - -

*Adjusted for age, gender, years living on the street, previous level of education, and reasons for being homeless.

Statistical significant odds ratios are boldfaced (*p* < 0.05).

## Discussion

The prevalence of substance use in this study is comparable to figures from other studies [4, 12] but lower than the rates found in other African countries [10, 34, 35]. In the past one month the sample reported 12.0% and 16.2% daily use of alcohol and marijuana respectively. Even though, these rates are relatively low, they suggest that these drugs are easily accessible to this population. Comparing this prevalence to studies in the general population, [36] reported 12.7% alcohol use and 5.2% among school going adolescents in Ghana. The high prevalence of marijuana use could be attributed to street socializing by peers on the street [37], and as coping mechanisms to reduce emotional problems [15]. The fact that approximately one in six of the youth reporting using marijuana shows the extent of risk youth face on the street of Accra.

The study also revealed that over 54% of the homeless youth reported having exchanged sex for food, money and even a place to sleep, with females more likely to engage in such behaviours than males. The high prevalence of females engaging in survival sex is consistent with studies conducted in developed countries [3, 22, 38] and those from sub-Saharan Africa including Ghana [5, 20, 25, 39, 40]. These findings also showed the vulnerability of females to exchange sex for money and or protection in a male dominated street life. A recent study in Ghana, described that insecurity on the street and the avoidance of forced sex, compelled females to seek protection from older boys by forming sexual relationships with them [5]. Power dynamics and females’ inability to generate income through other forms of work, has also seen as contributory factors that “push” female street youth to engage in survival sex [3]. It is however, important to note that an equally high number of males in this study also reported involvement in survival sex. Since over 54% of the sample had engaged in survival sex, implies that at least one out of every 2 homeless youth living on the street of Accra, are engaging in some form of prostitution.

The findings of the study showed that more female had smoked cigarette, used alcohol and marijuana than male in the past month. This finding contradicts previous studies among homeless youth where males generally reported higher substance use more than females [6, 9, 11, 13]. The sex difference may possibly be attributed to the fact that female adolescents with sexual abuse histories do abuse drugs on the street [37]. In this study, more females reported sexual abuse as a reason for being homeless than male participants. The extent of their engagement in survival sex is possibly linked to supporting their substance use behaviours. While early adolescents were more likely to abuse alcohol than the other age groups, marijuana use increases with age. Early adolescence as a stage of development is fueled with experimentation [41], and is likely to explain their vulnerability to experiment with alcohol which is generally accessible to both minors and adults in Ghana [42]. The increase of marijuana use with age is not surprising as it is less accessible as it is against the law and homeless youth have to develop various mechanisms to access it. This finding is also suggestive of the fact that the longer youth live on the street, the more likely they are to engage in substance use including marijuana [43], as the experiences of homelessness increases the vulnerability to substance use on the street [7, 12]. Social estrangement, which occurs when street youth who move to the street, become deep-rooted in street life with time, may explain youth susceptibility to engage in high risk behaviours including substance use [44]. Substance use with increased age may be associated with increased risk for the involvement in risky sexual behaviours.

A striking result in the study was that substance use among street youth particularly alcohol use, marijuana and cigarette smoking were independently associated with having unprotected sex, multiple sexual partners and engagement in survival. This aggregation of health damaging behaviours is consistent with the theory of problem behaviour [45]. The clustering of health risk behaviours among homeless youth is more enhanced as they are predisposed to several risk factors heightening multiple vulnerabilities [4, 11, 12]. Among street children in the Democratic Republic of Congo (DRC), a constellation of health compromising behaviours were reported where a history of drug use were linked to the engagement in sexual risk behaviour [11]. Also among Egyptian, Malawian and Sudanese street children, similar evidence of co-occurrence of health compromising behaviours has been identified [4, 39, 40]. Substance use is viewed as a risk factor to other health damaging behaviours, particularly risky sexual behaviour [46]. In the present study, it is unclear whether homeless youth engaged in risky sexual behaviour because they were under the influence of substance use or whether they engage in risky sexual behaviours as a means to support substance use or some “third factor” such as the social environment (e.g. peer influence and social networks) that facilitated risky sex or both behaviours. It is however known that substance use interfere with rational behaviours [8], which could make individuals more vulnerable to unsafe behaviours.

Health compromising behaviours among homeless youth in this study shows their vulnerability for HIV infection as a result of their high levels of sexual risk behaviours i.e. multiple sexual partners, engagement in survival sex and inconsistent condom use in contexts of considerably high alcohol and drug use. This linkage between substance use vulnerability to acquiring HIV and AIDS is well established [20, 25, 47].

The findings from this study have implications for future intervention efforts and research. First, the results suggest that over 10 percent of the homeless youth abuse substance such as alcohol and marijuana, and concurrently engaged in risky sexual behaviours. It is therefore important for agencies and NGOs who provide psychosocial services to homeless youth to have access to clinical psychologists and other mental health professionals who can assess, support, and treat the varied health problems such as substance use and risky sexual behaviours that have been shown to be associated with homelessness. This could be done in collaboration with the Government of Ghana through the Ministry of Gender, Children and Social Protection, that has oversight responsibilities among others to promote the rights of women and children. Second, the findings suggest a constellation of health risk behaviours and vulnerability among homeless youth, where substance use was associated with unprotected sex, having multiple sexual partners and commercialization of sex. This thus put homeless youth at risk for several physical and mental health problems such as HIV and STIs infection and drug dependency. It is therefore important for homeless youth service providers to address the effect of multiple risk behaviours through health promotion interventions that to address both substance use and risky sexual behaviours. Third, our findings reveal that substance use increases with age, which means that as homeless youth become older, they would be more likely to engage in substance abuse and abuse. It is therefore important for NGOs and international organizations that provide psycho-social services for homeless youth to target early entrants to the street before they become familiarized with the street sub-culture which is characterized with violence, substance abuse and sexual risky behaviours. This could be done by initiating harm reduction programmes that would address the needs of this population by ensuring that they use condoms correctly and consistently when engaging in sexual intercourse, especially for those who have multiple sexual partners. This initiative could use previously successful street youth to act as peer educators, as peer-to-peer contact has proven to be one of the effective approach to reaching most-at-risk young people [48]. Fourth, cognizance of gender dimensions is important in developing interventions for homeless population. A client-centered approach could be used through the development of trust when dealing with psycho-social problems, especially in respect of females who might have been sexually abused or involved in some form of survival sex. On the contrary, since males are known to be perpetrators of sexual violence on the street [33], programmes must focus on reducing or limiting such abuses. Finally, the multiplicity of problems experienced by homeless youth, demands a responsive mental health system. However, in Ghana the mental health system is not well developed, with accessibility for vulnerable groups such as homeless youth limited. Community-based organizations and NGOs who have taken on such responsibilities of providing limited psycho-social services have workers who might not receive the adequate training in a variety of psychotherapeutic approaches.

It should be noted that there as some limitations to this study. First, because it is impossible to randomly sample homeless youth from the population of homeless adolescents in Accra, Ghana, these data are not based on a probability sample and therefore results cannot be generalize to the whole population of homeless youth in Accra. Notwithstanding this, collecting data from the different places within Central Business District of Accra increased the reliability of the findings. Further research is however needed to determine whether homeless youth in other parts of Accra and or other regions of Ghana hold similar perspectives as found in this study. Secondly, results of the study should be interpreted cautiously as the study cross-sectional nature of the data limits causal inference. Third, the measures are based on homeless youth self-reports, which might be confounded by systematic bias and social desirability. However, if there was any reporting bias, the direction was under reporting rather than over reporting of the use substance use and sexual behaviour especially for male participants. Despite these shortfalls, on a whole, however, this study fills in an important gap in literature and provides useful piece of information for harm reduction interventions among homeless youth.

## Conclusion

This study examined the relationship between substance use and sexual risky behaviours among homeless youth. Substance use, especially alcohol and marijuana use among the sample in this study was relatively high and was used as a means of fitting in and coping with emotional distress. Substance use was found to be risk factor for risky sexual behaviours among Ghanaian homeless youth, placing them at risk for contracting STIs including HIV, with its accompanying health and social implications as well as bringing them into conflict with the laws. Harm reduction programmes targeting these youth must take in consideration the likelihood of substance use especially marijuana and alcohol use among the sexually experienced youth. Further studies are needed within the African context using stronger statistical models to explore the multiple pathways between substance use and risky sexual behaviours among homeless populations.
